# Developmental Factors That Predict Head Movement During Resting-State Functional Magnetic Resonance Imaging in 3–7-Year-Old Stuttering and Non-stuttering Children

**DOI:** 10.3389/fnins.2021.753010

**Published:** 2021-11-03

**Authors:** Chelsea A. Johnson, Emily O. Garnett, Ho Ming Chow, Gregory J. Spray, David C. Zhu, Soo-Eun Chang

**Affiliations:** ^1^Department of Communicative Sciences and Disorders, Michigan State University, East Lansing, MI, United States; ^2^Department of Psychiatry, University of Michigan, Ann Arbor, MI, United States; ^3^Department of Communication Sciences and Disorders, University of Delaware, Newark, DE, United States; ^4^Department of Radiology and Cognitive Imaging Research Center, Michigan State University, East Lansing, MI, United States

**Keywords:** temperament, stuttering, fMRI, children, pediatric MRI, neuroimaging, sex differences

## Abstract

Early childhood marks a period of dynamic neurocognitive development. Preschool-age coincides with the onset of many childhood disorders and is a developmental period that is frequently studied to determine markers of neurodevelopmental disorders. Magnetic resonance imaging (MRI) is often used to explore typical brain development and the neural bases of neurodevelopmental disorders. However, acquiring high-quality MRI data in young children is challenging. The enclosed space and loud sounds can trigger unease and cause excessive head movement. A better understanding of potential factors that predict successful MRI acquisition would increase chances of collecting useable data in children with and without neurodevelopmental disorders. We investigated whether age, sex, stuttering status, and childhood temperament as measured using the Child Behavioral Questionnaire, could predict movement extent during resting-state functional MRI (rs-fMRI) in 76 children aged 3–7 years, including 42 children who stutter (CWS). We found that age, sex, and temperament factors could predict motion during rs-fMRI scans. The CWS were not found to differ significantly from controls in temperament or head movement during scanning. Sex and age were significant predictors of movement. However, age was no longer a significant predictor when temperament, specifically effortful control, was considered. Controlling for age, boys with higher effortful control scores moved less during rs-fMRI procedures. Additionally, boys who showed higher negative affectivity showed a trend for greater movement. Considering temperament factors in addition to age and sex may help predict the success of acquiring useable rs-fMRI (and likely general brain MRI) data in young children in MR neuroimaging.

## Introduction

Though generally considered to be a technique that is difficult to apply in young children, MRI has been used at an increasing rate over the last few decades to study brain development in typically developing children and children with neurodevelopmental disorders (Price, 2012). The onset of many neurodevelopmental disorders, such as dyslexia (Raschle et al., 2011; Cao et al., 2018; Wang et al., 2018), attention-deficit/hyperactivity disorder (ADHD; Kieling and Rohde, 2010), specific language impairment (SLI), autism spectrum disorders (ASD), intellectual disability (Jacola et al., 2014), and developmental stuttering (Bloodstein and Bernstein Ratner, 2008; Yairi and Ambrose, 2013) occurs at preschool-age or earlier, typically defined as between 3 and 5 years of age (CDC, 2021). The ability to detect subtle neural differences near symptom onset can be greatly enhanced by the acquisition of whole-brain measures using functional magnetic resonance imaging (fMRI), which is crucial to extract both localized and network-level information to expand our understanding of the nature of these neurodevelopmental disorders. Early detection of brain network-level changes associated with childhood neurodevelopmental disorders might pave the way for developing assessments, interventions, and better prognostic markers for these disorders; however, this requires acquiring MRI data from very young children.

Collecting high-quality MRI data from preschool-age and young school-age children is challenging (for a discussion, see Greene et al., 2016, 2018; Copeland et al., 2021). Young children may exhibit fear or anxiety associated with the scanning environment, which can lead to increased movement during scanning. Previous research has suggested that scan duration, movement restrictions, being placed within a confined space, and the loud noise during scanning may contribute to a child having difficulty completing a scan (Marshall et al., 1995; Tyc et al., 1995, 1997). Potential modulatory factors that have been investigated in clinical disorders and typically developing children include age and sex; however, the contribution of these variables is understudied and thus poorly understood (Malisza et al., 2010; Cahoon and Davison, 2014). There is no strong evidence for an effect of sex (Malisza et al., 2010; Cahoon and Davison, 2014); however, not all studies explicitly examined sex as a factor (Yerys et al., 2009). These studies importantly did not examine the differences between sexes within clinical populations, despite a large body of evidence suggesting that different behaviors and developmental patterns are present among boys and girls diagnosed with neurodevelopmental disorders (e.g., Gaub and Carlson, 1997; Hartley and Sikora, 2009; Mandy et al., 2012; Arnett et al., 2013).

Age is consistently found to influence the “success” of MRI data collection in children, which can be defined as data that are relatively free of movement artifacts and are thus of sufficient quality to be included in data analysis. Age has been shown to influence data quality across various data collection methods in terms of compliance, active participation, and reliability of measurement (Yerys et al., 2009; Malisza et al., 2010). For MRI in particular, it is difficult for young children to remain still for the 30–60 min scanning period required for most research scans (Yerys et al., 2009; Malisza et al., 2010). Previous research has shown that for neuroimaging studies in children 4–6 years of age, researchers may need to increase recruitment for data collection by 20–40% to obtain sufficient useable data without movement artifacts (Yerys et al., 2009). Malisza et al. (2010) studied a multi-step MRI preparation protocol that involved desensitization to the MRI environment up to and including entering the bore, but not subsequent scanning. Results indicated that children 2–3 years of age have a significantly lower rate of successful completion of the approach sequence than children 6–7 years of age: 2–5-year-olds had a failure rate of 50%, while 6–7-year-olds only had a failure rate of 35%. These authors also suggest that researchers should anticipate a failure rate of at least 50% if a study includes children 2–7 years of age (Malisza et al., 2010). The sample sizes in this study differed across age groups however, potentially influencing their results.

Still, some young children seem to be able to tolerate scanning better than much older children. This observation suggests that interactions with variables other than age may predispose some children to tolerate scanning better than others. Given the challenges researchers face in acquiring MRI data in young children, it is vital to understand factors that may interact with the age of the child that could lead to excessive movement during scanning. One such factor that may influence pediatric scanning success is temperament, or one’s innate behavioral dispositions. For example, temperament affects a child’s tolerance to many clinical environments such as doctor and dental visits (e.g., Pate et al., 1996; Klingberg and Broberg, 1998; ten Berge et al., 1999). To date, few studies have directly investigated temperament as it relates to the successful completion of MRI scanning in children (Voepel-Lewis et al., 2000; Cahoon and Davison, 2014). From these studies, the best predictors of movement during scanning were poor attention and adaptability skills (i.e., ability to handle novel experiences) among preschool- and school-age children (Voepel-Lewis et al., 2000; Cahoon and Davison, 2014). Children with neurodevelopmental disorders may display decreased attention regulation and adaptability, which might further compromise their ability to tolerate scanning (Lufi and Parish-Plass, 1995; McIntosh and Cole-Love, 1996; White, 1999; Tillman et al., 2003; Hepburn and Stone, 2006; Konstantareas and Stewart, 2006; Yoo et al., 2006; Cho et al., 2008; Melegari et al., 2015). While some studies have shown that children with neurodevelopmental disorders such as ADHD, and ASD, or developmental delays tend to have fewer successful scans or lower test-retest reliability of fMRI metrics than controls (Somandepalli et al., 2015), the reasons for this have not been clarified, especially in young children (Yerys et al., 2009; Cahoon and Davison, 2014). Moreover, temperament factors such as adaptability and attentional control develop differently between the sexes, even in typically developing children. Preschool-age girls tend to have more advanced skills related to effortful control than their age-matched male peers (Kochanska et al., 2000). If temperament factors that influence MRI scanning develop more slowly in boys than girls, boys with neurodevelopmental disorders may have an even more challenging time tolerating MRI scanning.

Several studies to date have reported that children who stutter (CWS) and children who do not stutter differ in some temperament dimensions. CWS have also been shown to demonstrate decreases in inhibition (Embrechts et al., 2000; Eggers et al., 2010; Felsenfeld et al., 2010; Eichorn and Pirutinsky, 2021), attentional shifting (Eggers et al., 2010; Eichorn and Pirutinsky, 2021), attentional focusing (Embrechts et al., 2000; Rocha et al., 2019; Eichorn and Pirutinsky, 2021), and attentional regulation (Karrass et al., 2006; Eichorn and Pirutinsky, 2021). They also exhibit increased difficulty adapting to change (Anderson et al., 2003; Howell et al., 2004), present with greater negative affect (Howell et al., 2004; Eggers et al., 2010; Ntourou et al., 2013), display increased emotional reactivity (Karrass et al., 2006), show higher activity levels (Embrechts et al., 2000; Howell et al., 2004), demonstrate more anger and frustration (Eggers et al., 2010; Rocha et al., 2019), and exhibit more impulsivity (Embrechts et al., 2000). Because these factors may contribute to tolerance of the scanning procedures in clinical populations, information that further elucidates relationships between temperament factors, age, sex, presence of a clinical condition (in this case, stuttering), and head movement during scanning, could help develop strategies to increase chances of collecting usable MRI data that is comparable across the clinical and control groups. If temperamental factors associated with poor attention and adaptability skills influence scanning tolerance in children, those with neurodevelopmental disorders may already be at a greater disadvantage and have a lower probability of success, leading to spurious group differences observed during data analysis that may be erroneously attributed to the core clinical condition.

The current study investigated whether a diagnosis of developmental stuttering, age, sex, and temperament as assessed by scores on the Child Behavior Questionnaire (CBQ; Rothbart et al., 2001), could predict excessive head movement during resting-state fMRI scanning in young children who stutter and their non-stuttering peers (hereafter referred to as “controls”). We focused our temperament analyses on the three CBQ composite scores: effortful control, negative affectivity, and extraversion/surgency. Effortful control reflects a child’s ability to control attentional processes and regulate behaviors, such as their ability to maintain focus on a task. Negative affectivity measures children’s negative emotional responses, such as a negative response to an adverse, unique, or high-intensity event. Extraversion/surgency describes how extroverted or outgoing a child may be, for example, how willing a child may be open to new experiences (Rothbart et al., 2011). Guided by previous research reporting temperament differences between CWS and controls as well as the relationship between temperament and MRI tolerance in children, we tested the following hypotheses. First, we expected that CWS would exhibit significantly more movement during scanning than controls. Second, we expected that the groups would differ in temperament indices that may affect tolerance of MRI procedures, including effortful control and negative affectivity, because of their relationship to poor attention and adaptability skills (i.e., ability to handle novel experiences) that have been shown to be associated with increased tolerance of MRI scanning (Voepel-Lewis et al., 2000; Cahoon and Davison, 2014). These factors are consistent with the effortful control scale on the CBQ that measures self-regulation of behavior and emotions as well as negative affectivity, which is associated with a child’s negative response to adverse or high-intensity events. Our third hypothesis tested whether the extent of movement during MRI scanning is influenced by temperamental characteristics associated with lower adaptability skills and attentional control, such as low effortful control and higher negative affectivity scores. We hypothesized that these temperamental differences would be associated with the most movement artifacts in their scans and that this relationship would be modulated by age and sex of the child.

## Materials and Methods

### Participants

This current study included 76 participants (42 CWS, 34 controls) from an ongoing longitudinal project examining the neural bases of developmental stuttering (Chow and Chang, 2017). MRI data collected from the first visits were used in this study from the larger longitudinal study. Details on recruitment, testing, and in/exclusion criteria for this longitudinal study can be found in Chang and Zhu (2013). Briefly, all children were monolingual English speakers and scored within two standard deviations of the mean on all standardized speech-language assessments and intelligence tests. Participants included in this study were those with complete CBQ scores and resting-state fMRI (rs-fMRI) datasets collected. The age range of participants was 37–86 months (3–7 years) at the time of scanning (*M* = 4.8 years, *SD* = 1.2 years). See Table 1 for participant demographic information. Most (*n* = 60; 79%) of the children were considered within the preschool age (3–5 years).

**TABLE 1:** Demographic information of children who stutter (CWS) and controls included in this study.

	CWS, *n* = 42 (15 girls)		Controls, *n* = 34 (19 girls)		Between group comparisons	
	Mean (*SD*)	Range	Mean (*SD*)	Range	*t*	*p*
Age (months)	59.09 (14.89)	38–86	57.10 (13.50)	37–83	–0.611	0.543
Socioeconomic status^a^	6.12 (0.769)	5–7	6 (0.975)	4–7	–0.571	0.570
Head movement during scanning^b^	29.60 (27.08)	0–93	28.95 (24.00)	0–84.14	–0.112	0.911
Negative affectivity	3.73 (0.71)	1.83–5.12	3.93 (0.74)	2.22–5.32	1.185	0.242
Surgency	4.64 (0.70)	2.52–6.05	4.87 (0.75)	3.13–6.36	1.354	0.180
Effortful control	5.23 (0.47)	4.22–6.11	5.11 (0.73)	3.37–6.79	645.0^c^	0.471

*^a^Maternal education level.*

*^b^Percentage of volumes with FD > 0.5 mm.*

*^c^Mann-Whitney U-test used due to not meeting assumptions for normality.*

All study procedures were approved by the Institutional Review Board at Michigan State University’s in accordance with the Declaration of Helsinki. Informed consent/assent was obtained from all children and their parents before participation. Children were given small prizes and stickers for participation and received nominal remuneration.

### Behavioral Testing

#### Speech and Language Testing

All children completed a battery of cognitive and speech-language assessments, as detailed in Chang and Zhu (2013). Briefly, these tests included the Clinical Evaluation of Language Fundamentals Preschool-2 (CELF-P2; Wiig et al., 2004) or Clinical Evaluation of Language Fundamentals-5 (CELF-5; Wiig et al., 2013), the Goldman-Fristoe Test of Articulation-2 (GFTA-2; Goldman and Fristoe, 2000), and the Wechsler Scales of Intelligence for the participants’ appropriate age group (Wechsler, 1999, 2003). Regardless of group, all children scored within the typical range for their age on these standardized assessments. Consistent with previous studies, a child was considered to be stuttering if they met the following criteria: (1) three or more stuttering-like disfluencies per 100 syllables, (2) a diagnosis of “very mild” or greater based on the index score from the Stuttering Severity Instrument (SSI-4; Riley, 2009) during a narrative story-telling task and a conversation sample collected with a certified speech-language pathologist (Ambrose and Yairi, 1999), and (3) expressed concern from parent and clinician impression consistent with stuttering diagnosis.

#### Measures of Temperament

Parents completed the 94-item CBQ (Rothbart et al., 2001), which assesses the child’s temperament within the previous 6 months. This questionnaire uses a 6-point Likert scale to evaluate children between the ages of 3–8 years (Putnam and Rothbart, 2006). Responses to each of the 94 questions are then grouped to form 15 subscales, including *activity level, anger/frustration, approach, attentional focusing, discomfort, falling reactivity and soothability, fear, high-intensity pleasure, impulsivity, inhibitory control, low-intensity pleasure, perceptual sensitivity, sadness, shyness*, and *smiling and laughter* (for a description of each variable, see Rothbart et al., 2001). These aforementioned subscales are used to calculate three CBQ composite scores were calculated: effortful control, negative affectivity, and extraversion/surgency. These measures were used in the statistical analyses described below.

#### Magnetic Resonance Imaging Data Acquisition and Head Motion Measures

Before the MRI visit, all children underwent an MRI training protocol that involved desensitization to the sights and sounds of the scanner environment using games and by visiting a mock MRI scanner. An MRI training protocol that was developed and tested for young children as reported in Theys et al. (2014) was implemented to ensure that all children were ready and willing to participate in the subsequent MRI scanning session. On the day of the MRI scan, a trained clinician or graduate student sat next to the child at all times during the scanning session to monitor the child for excessive movement or distress, at which point scanning was stopped and restarted as tolerated. All scans were acquired using a 3T GE Signa HDx MR scanner (GE Healthcare) with an 8-channel head coil in the Department of Radiology at Michigan State University. Each scan session included a 10-min rs-fMRI scan, which generally occurred after the structural MRI (3D IRFSPGR) and diffusion tensor imaging (DTI) scans (45 min total). The rs-fMRI images were collected with echo-planar imaging using the following parameters: 38 contiguous 3-mm axial slices in an interleaved order, echo time = 27.7 ms, repetition time = 2,500 ms, flip angle = 80°, field of view = 22 cm, matrix size = 64 × 64, ramp sampling, with the first four data points discarded, and each volume of slices acquired 164 times (Chang and Zhu, 2013). Each rs-fMRI scan was co-registered to the first volume using rigid body rotation. The movement parameters obtained in the co-registration step were used to calculate the frame-wise displacement (FD), an estimate of volume-to-volume head movement (Power et al., 2012) using AFNI’s 1d_tool.py and 1deval. For each participant, the percentage of volumes with FD > 0.5 mm was used to determinate the degree of excessive head motion. This threshold was selected because it has been used in other previous studies that have examined movement during scanning (Power et al., 2012, 2014; Xu et al., 2018). A standard FD threshold is not established in the field and thus the selection of 0.5 mm as the FD threshold for our analyses was guided by the above sources. However, we also explored results using FD thresholds of 1, 0.7, and 0.3 mm, which are presented in Supplementary Tables 1–15 for readers who are interested in the effects of different FD thresholds. We also note that FD was used as a measure of movement because it is a direct measure of head movement compared to other methods like DVARS, which is an indirect measure of movement.

### Data Analysis

#### Between-Group Differences

All statistical analyses were run using IBM SPSS Statistics, version 26. We first used separate independent samples *t*-tests to compare differences between CWS and controls in (a) movement during rs-fMRI scanning and the three CBQ composite scores: (b) effortful control, (c) negative affectivity, and (d) surgency. The effortful control variable did not meet the assumption of homogeneity of variances, as tested by Levene’s Test for Equality of Variances (*F* = 10.77, *p* = 0.001), so a Mann-Whitney *U*-test was used to compare CWS and controls for this variable.

#### Regression Analyses

A linear regression analysis was used to further examine group differences in movement during rs-fMRI scans with age and sex entered as covariates. Due to stuttering status being a non-significant predictor in this model (Table 2) and the lack of difference in movement in children who stutter and controls (Table 1), the two groups were collapsed for the subsequent analyses. First, a linear regression assessed the effect of age and sex on head movement during rs-fMRI (Table 3). Additionally, three separate linear regression analyses were conducted to assess the effects of each of the three temperament variables as well as age and sex on head movement during rs-fMRI scanning (Tables 4–6). Lastly, regression models that examined the effects of temperament were conducted separately in girls and boys (Table 7). Individual movement data were transformed using a square root transformation due to not meeting normality of residuals for all regression analyses. All movement analyses were performed using the transformed data.

**TABLE 2:** The effects of sex, age, clinical status (stuttering, control), and their interactions on head movement.

	Effect	*B*	*SE*	*t*	*p*
*Movement*	Intercept	3.779	0.543	6.959	< 0.001*
	Sex	1.836	0.735	2.498	0.015*
	Age	–0.071	0.034	–2.108	0.039*
	Group	0.936	0.831	1.127	0.264
	Sex*Age	0.026	0.039	0.660	0.511
	Age*Group	–0.013	0.039	–0.336	0.738
	Sex*Group	–1.642	1.113	–1.475	0.145

*Significant at p < 0.05.

**TABLE 3:** The effects of age, sex, and their interactions on head movement (groups combined).

	Effect	*B*	*SE*	*t*	*p*
Movement	Intercept	4.170	0.406	10.267	< 0.001*
	Age*Sex	0.021	0.039	0.535	0.594
	Sex	1.124	0.549	2.058	0.043*
	Age	–0.072	0.028	–2.623	0.011*

*Significant at p < 0.05.

**TABLE 4:** Regression results examining effects of Surgency, Age, and Sex and interactions between Sex*Surgency and Age*Surgency.

	Effect	*B*	*SE*	*t*	*p*
Movement	Intercept	5.528	2.344	2.358	0.021*
	Surgency	–0.056	0.475	–0.118	0.907
	Age	0.028	0.124	0.228	0.821
	Sex	–1.474	3.838	–0.384	0.702
	Sex*Surgency	0.087	0.805	0.109	0.914
	Age*Surgency	–0.019	0.026	–0.737	0.464

*Significant at p < 0.05.

**TABLE 5:** Regression results examining effects of Effortful Control, Age, Sex, and interactions.

	Effect	*B*	*SE*	*t*	*p*
Movement	Intercept	11.612	2.659	4.368	< 0.001*
	Effortful control	–1.263	0.526	–2.403	0.019*
	Age	–0.043	0.153	–0.280	0.780
	Sex	–11.290	5.179	–2.180	0.033*
	Sex*Effortful control	1.980	0.983	2.015	0.048*
	Age*Effortful control	–0.004	0.029	–0.153	0.879

*Significant at p < 0.05.

**TABLE 6:** Regression results examining effects of Negative Affectivity, Age, Sex, and interactions.

	Effect	*B*	*SE*	*t*	*p*
Movement	Intercept	1.414	1.963	0.720	0.474
	Negative affectivity	0.986	0.493	1.999	0.050*
	Age	–0.135	0.118	–1.137	0.260
	Sex	5.538	2.988	1.853	0.068
	Sex*Negative affectivity	–1.716	0.760	–2.258	0.027*
	Age*Negative affectivity	0.017	0.030	0.563	0.576

*Significant at p < 0.05.

**TABLE 7:** Regression results examining effects of age and temperament in boys and girls.

		Girls				Boys			
	Effect	*B*	*SE*	*t*(31)	*p*	*B*	*SE*	*t*(39)	*p*
Movement	(Intercept)	11.06	2.61	4.24	< 0.001*	5.53	2.23	2.47	0.018*
	Negative affectivity	–0.70	0.54	–1.30	0.203	0.91	0.51	1.78	0.082
	Age	–0.07	0.02	–2.81	0.008*	–0.06	0.029	–2.26	0.029*
Movement	(Intercept)	8.09	2.95	2.73	0.010*	8.83	3.03	2.91	0.006*
	Surgency	0.06	0.62	0.11	0.913	–0.10	0.48	–0.21	0.829
	Age	–0.07	0.02	–2.66	0.012*	–0.05	0.02	–1.80	0.079
Movement	(Intercept)	4.66	4.24	1.09	0.281	14.79	3.21	4.60	< 0.001*
	Effortful control	0.73	0.77	0.94	0.353	–1.24	0.53	–2.32	0.025*
	Age	–0.07	0.02	–2.88	0.007*	–0.05	0.02	–2.08	0.043*

*Significant at p < 0.05.

## Results

### Between-Group Differences

Children who stutter and controls did not differ significantly in age, socioeconomic status, movement during rs-fMRI scanning, negative affectivity, surgency, or effortful control (Table 1). Multiple linear regression was used to examine the effects of age, sex, stuttering status, and their interactions on head movements. Age and sex were both significant predictors of head movement (Table 2). Age was negatively associated with head movement; not surprisingly, older children tended to move less than their younger peers. Boys tended to have more volumes with significant movement than girls in this model. However, stuttering status (i.e., group) was not significantly associated with head movement, even when accounting for age and sex. Therefore, data from the two groups of children were combined for subsequent analyses.

### Relationship Between Head Movement, Age, and Sex

Multiple linear regression examined the effects of age, sex, and their interactions on head movement. The regression equation was significant [*F*(3) = 5.43, *p* = 0.002], with an adjusted R^2^ of 0.151. Both age and sex were significant predictors of head movement in the model (Table 3). Age was negatively associated with head movement. Boys had more volumes with significant movement than girls in the study.

### Relationship Between Temperament and Head Movement

**FIGURE 1:**
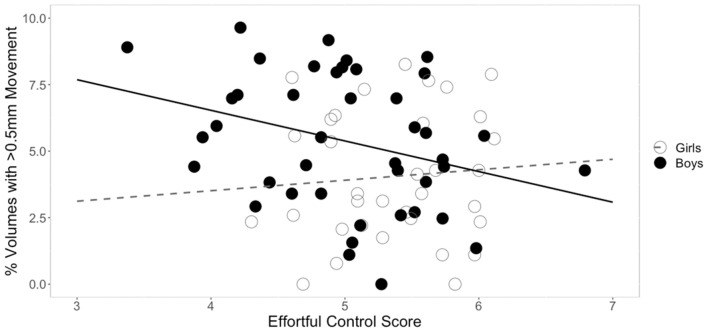
Sex differences in the relationship between effortful control and movement. The square root of percent of volumes with greater than 0.5 mm of movement are plotted on the *y*-axis. For boys, effortful control was negatively correlated with movement. This relationship was not significant in girls.

**FIGURE 2:**
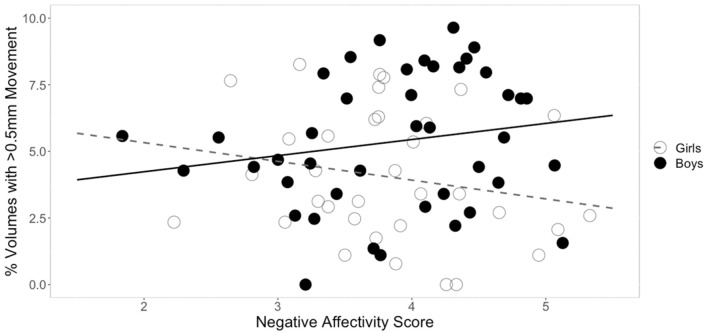
Sex differences in the relationship between negative affectivity and movement. The square root of percent of volumes with greater than 0.5 mm of movement are plotted on the *y*-axis. While there was a significant interaction between negative affectivity and sex, follow-up sex specific regressions models showed that the relationship between negative affectivity scores and head movement were not significant in either sex.

Three separate multiple linear regression analyses were conducted to examine whether the three CBQ composite variables (surgency, effortful control, negative affectivity) predicted head movement. Each regression model included five predictors: main effect of the CBQ variable, sex, age, the interaction between age and the CBQ variable, and the interaction between sex and the CBQ measure (Tables 4–6 and Figures 1, 2). The three-way interaction between age, sex, and CBQ variable was not included because the interaction between age and sex was not significant in the analysis presented in Table 2. The first model examined the CBQ variable surgency. The overall model was significant [*F*(5) = 3.250, *p* = 0.011, adjusted *R*^2^ = 0.130]; however, none of the predictors were significant (Table 4). The second model examining the effect of effortful control was significant [*F*(5) = 4.691, *p* = 0.001, adjusted *R*^2^ = 0.197]. Effortful control, sex, and the interaction between sex and effortful control were the significant predictors of movement in the model (Table 5). The significant interaction term between effortful control and sex indicates that the relationship between effortful control and movement extent varies by the sex of the child; therefore, two additional exploratory regressions models were run, separately in boys and girls. These analyses showed that there was a significant relationship between effortful control and head movement in boys, such that boys with greater effortful control scores moved less during scanning, as shown in Figure 1 and Table 7. In contrast, no significant relationship between these two variables was found in girls. The third model predicting movement based on negative affectivity was also significant [*F*(5) = 4.459, *p* = 0.001, adjusted *R*^2^ = 0.187]. While sex alone was not a good predictor of movement, negative affectivity and its interaction with sex were significant predictors of movement (Table 6). Separate analyses for boys and girls showed that negative affectivity did not predict more head movement in either sex (Table 7 and Figure 2).

## Discussion

The present study examined the effects of clinical group (stuttering status), age, sex, and temperament on the extent of head movement during resting-state functional magnetic resonance imaging (rs-fMRI) scanning in young children. We predicted that children who stutter (CWS) would exhibit more movement during scanning than the control group, as measured by percent of total MRI volumes that contained head movement exceeding 0.5 mm. Instead, results indicated that stuttering status was not a significant predictor of movement, and CWS did not differ from controls in any temperament score measured by the CBQ (Tables 1, 2). Hence, we collapsed the groups for our subsequent analyses examining the predictive effect of temperament on movement. Before discussing the significant results from the combined analyses, we first discuss possible reasons for the lack of significant group differences in movement or temperament between children who stutter and controls.

### Children Who Stutter Do Not Differ From Controls in Movement or Temperament

The present findings do not support significant differences between CWS and controls on temperament scores as measured by the CBQ. While previous research reported differences between CWS and their control peers on various tasks or instruments that measure temperament (for a review, see Conture et al., 2013), other studies have failed to find differences between these groups (Reilly et al., 2013; Kefalianos et al., 2017; Walsh et al., 2019; Eggers et al., 2021). It is important to note that the studies of temperament in CWS vary greatly in their methods of sampling (e.g., the children recruited through a clinic vs. from a community sample), age range, and as pointed out by Alm (2014), sample sizes as well as the specific test to measure temperamental differences. Unlike what was done in the present study, which recruited both CWS and control groups from the larger community and not relying on clinical samples, many studies recruit CWS from speech and language clinics (e.g., Anderson et al., 2003; Karrass et al., 2006; Eggers et al., 2010; Felsenfeld et al., 2010) and would have included children currently undergoing therapy. Those CWS who are recruited from clinics may be more likely to present with comorbid clinical disorders such as attention deficit hyperactivity disorder, language delays, and affective disorders. In addition, young CWS who seek therapy may be those that tend to exhibit higher negative reactions to stuttering than those who do not seek treatment for their stuttering. Thus, CWS who are recruited primarily from clinics have a higher likelihood of differing in temperament measures relative to those that do not seek treatment. Currently, it is difficult to compare the results of this study to others that used differing recruiting methods and collected data from older and/or wider age ranges (Eggers et al., 2010; Felsenfeld et al., 2010; Rocha et al., 2019) or studies using different temperament measurements than the CBQ used in this study (Anderson et al., 2003; Howell, 2004; Karrass et al., 2006; Felsenfeld et al., 2010; Ntourou et al., 2013). More studies are needed to confirm whether temperament factors differ between CWS and controls, taking into account varied methods of sampling procedures.

### Temperament Factors Predict Head Movement Differently in Boys Compared to Girls

Not surprisingly, age was a significant predictor of movement. However, age was no longer a significant predictor of movement when temperament factors were entered in the statistical model. Based on previous findings from studies that examined movement during MRI in children (e.g., Yerys et al., 2009), we predicted that temperament would be associated with movement during rs-fMRI. We found that the CBQ variables effortful control and negative affectivity predicted head movement from these analyses (Figures 1, 2). Both effortful control and negative affectivity had significant sex interactions, suggesting that the effects of effortful control and negative affectivity on movement are different in boys and girls (Tables 5–6). Our follow up analyses showed a significant negative correlation between *effortful control* and movement was observed for boys but not in girls (Table 7 and Figure 1). Boys with higher effortful control scores, reflecting better regulation of attentional resources during scanning, moved less during rs-fMRI procedures. In contrast, boys who showed higher *negative affectivity* showed a trend for greater movement. We now discuss effortful control and negative affectivity separately.

The difference of maturity rates in some effortful control skills between boys and girls in this study may have influenced the disparate relationship between effortful control and head movement among the two sexes in this study (for a discussion, see Viddal et al., 2015). Boys tend to have a more protracted developmental trajectory for effortful control than girls (e.g., Else-quest et al., 2006; Posner et al., 2007; Mutlu et al., 2013). Another possible explanation for the pattern of difference between sexes is that effortful control mediates the development of externalizing behavior in boys but not girls (e.g., Coe et al., 2020). These previous findings may explain the observed significant relationship between effortful control and movement in boys but not girls in this study. For example, girls may be able to comply with rs-fMRI procedures regardless of their effortful control scores.

While negative affectivity was a significant predictor of movement, and sex modulated this relationship (Table 6), the overall statistical significance of this finding was weak. Further, it was not a significant predictor of movement for either boys or girls when they were examined separately (Table 7). The data show a trend for more movement in boys with higher negative affectivity scores (Table 7 and Figure 2). This result suggests that boys who generally experience higher levels of negative affectivity were less able to tolerate scanning. Additionally, the significant interaction between sex and negative affectivity suggests that the relationship between negative affectivity and movement in girls is different from that of boys (Table 6). Negative affectivity is related to feelings of distress during unique or high-intensity experiences. Higher CBQ negative affectivity scores in children in this study could indicate a higher negative response to the novelty of the scanning environment, which may have, in turn, translated into increased movement. Previous studies have reported that higher levels of negative affectivity in childhood are associated with more externalizing problems, such as behaviors that are seen as disruptive or problematic, in childhood and later in development as teenagers (Eisenberg et al., 2001; Honomichl and Donnellan, 2012).

### Implications and Future Directions for Pediatric Imaging in Typically Developing and Clinical Populations

Although findings from the current study were based on rs-fMRI data quality, they can be applied to brain MRI studies in general. These findings have important implications for conducting MRI research in clinical populations. Previously it has been reported that clinical pediatric populations exhibit more head movement during scanning than controls. This pattern was not observed for the CWS in our study, who did not exhibit significant differences relative to control children in terms of head movement during rs-fMRI. Age, sex, effortful control, and to a lesser degree negative affectivity, were the factors found to be significantly associated with head movement during rs-fMRI in our study. This result suggests that some clinical populations, including children who stutter, may exhibit comparable tolerance to rs-fMRI procedures to their peers.

Further research is needed to better understand what factors may allow one clinical group to tolerate scanning compared to their peers. Children with autism often exhibit heightened sensitivity to their environment, especially for visual and auditory stimuli (for a discussion, see Stiegler and Davis, 2010), which may predispose this group to have more difficulties tolerating the loud sounds of the MRI machine and restrictions to movement. Results from the present study suggest that exploring other factors like temperament is valuable, as characteristics associated with developmental disorders are heterogeneous even within the same diagnostic category. For example, effortful control has been connected to a lower prevalence of behaviors affiliated with autism (e.g., Konstantareas and Stewart, 2006). More research is needed to understand which behavioral characteristics, including temperament, may predict scanning success across different neurodevelopmental disorders.

Findings from our study corroborate previous reports that have emphasized the importance of allowing young children to desensitize to the sights, sounds, and MRI environment. While several previous studies have indicated that the introduction of mock MRI practice sessions before the experimental session may significantly reduce potential movement and data loss during MRI scanning (Carter et al., 2010; de Bie et al., 2010; Woods-Frohlich et al., 2010; Barnea-Goraly et al., 2014; Theys et al., 2014; Thieba et al., 2018), few have examined how individual differences within pediatric populations may need to be considered when designing MRI desensitization interventions. It is essential to consider what steps researchers and clinicians can take to ensure the comfort of children and the chances of successful data collection carefully based on the needs of the child. Considering how individualized factors may influence a child, such as their age, sex, and temperament, may help those scanning pediatric populations decide what kind of intervention may be needed.

### Limitations

The data from this study were collected from a longitudinal study of developmental stuttering. These results may be less generalizable to children scanned for purely clinical purposes. For example, our study participants may have certain temperamental characteristics associated with willingness to volunteer in a research study, and in an MRI study in particular. Therefore, due to potential self-selection biases, our study may be most relevant to research environments where children volunteer to participate. Future studies involving larger sample sizes, as well as diversity in the pediatric neurodevelopmental populations explored in the study, are necessary to replicate and expand the findings reported in this study and understand how different conditions may predispose children to tolerate rs-fMRI scanning.

Our interpretation of how temperament affects movement during scanning is discussed within the context of previous research that explored disruptive externalizing behaviors, such as those that would get a child in trouble at school or warrant psychological evaluation and intervention. These conditions are quite different from the strict procedures involved in rs-fMRI scanning, which can be challenging even for adults. In this study, excessive movement was defined by volumes that contained movement greater than 0.5 mm. This value was chosen as a stringent movement parameter; however, research teams may be able to accept more or less movement depending on their analyses. This stringent threshold leaves a very small margin for error for these participants. Therefore, an inability to comply with these procedures should not be compared to behaviors of clinical significance or any typical behaviors that may be disruptive to everyday life.

Finally, several of the significant findings regarding how temperamental factors affect movement were near our *a priori* alpha level of 0.05. In particular, negative affectivity only shows a significant relationship with movement in the 0.5 mm analyses, but not at the other thresholds reported in Supplementary Tables 5, 10, 15. Therefore, caution is needed when interpreting our results in the context of previous research. While we argue that the three temperament factors are separate constructs, further confirmatory studies are needed to fully understand the nuanced relation between temperament and movement during scanning, especially in young children.

## Conclusion

This study examined whether developmental factors such as age, sex, specific temperament variables, and presence or a clinical diagnosis (stuttering) could predict head movement during MRI scanning in preschool-age and young school-age children. We found that in this sample of young children (3–7 years) who underwent rs-fMRI scanning for research purposes, age, sex, and temperament were predictors of motion during rs-fMRI scans. Children who stutter did not differ significantly from typical peers in head motion during rs-fMRI scans. Age and sex were good predictors of movement during rs-fMRI scanning when temperament factors were not considered. When examining temperament, the best potential predictor of head movement during scanning was effortful control and to a lesser degree negative affectivity. Results from this study suggest that assessing temperament factors may help identify children who could benefit from additional time to desensitize to the MRI environment and scanning procedures. Ultimately, this may lead to improved quality and increased quantity of useable brain scans acquired from young children, which is crucial to furthering our understanding of brain development in children with and without neurodevelopmental disorders.

## Data Availability Statement

The raw data supporting the conclusions of this article will made available by the authors upon reasonable request.

## Ethics Statement

The studies involving human participants were reviewed and approved by the Michigan State University Human Research Protection Program. Written informed consent to participate in this study was provided by the participants’ legal guardian/next of kin.

## Author Contributions

CJ, GS, S-EC, and HC: conceptualization. HC, S-EC, EG, CJ, GS, and DZ: methodology, writing—review, and editing. HC, EG, and CJ: formal analysis and investigation. CJ, S-EC, and EG: writing—original draft preparation. S-EC: funding acquisition. S-EC, EG, and HC: supervision. All authors contributed to the article and approved the submitted version.

## Conflict of Interest

The authors declare that the research was conducted in the absence of any commercial or financial relationships that could be construed as a potential conflict of interest.

## Publisher’s Note

All claims expressed in this article are solely those of the authors and do not necessarily represent those of their affiliated organizations, or those of the publisher, the editors and the reviewers. Any product that may be evaluated in this article, or claim that may be made by its manufacturer, is not guaranteed or endorsed by the publisher.

## Abbreviations

ADHD: Attention-Deficit/Hyperactivity Disorder
ASD: Autism Spectrum Disorder
CBQ: Child Behavior Questionnaire
CELF-5: Clinical Evaluation of Language Fundamentals-5
CELF-P2: Clinical Evaluation of Language Fundamentals Preschool-2
CWS: Children who Stutter
DTI: Diffusion Tensor Imaging
EEG: Electroencephalography
FD: Frame-Wise Displacement
Fnirs: Functional Near-Infrared Spectroscopy
MRI: Magnetic Resonance Imaging
rs-fMRI: Resting-State Functional Magnetic Resonance Imaging
SLI: Specific Language Impairment.
