# Supplementary material for: Developmental Factors That Predict Head Movement During Resting-State Functional Magnetic Resonance Imaging in 3–7-Year-Old Stuttering and Non-stuttering Children
Source: Front Neurosci. 2021 Nov 3;15:753010. doi: 10.3389/fnins.2021.753010 (PMC8595248; doi:10.3389/fnins.2021.753010)
Supplement: Supplementary file 1 [file Data_Sheet_1.docx]

**1mm results**

**Table S1**: The effects of sex, age, clinical status (stuttering, control) and their interactions on head movement.

|  | **Effect** | **B** | **SE** | **t** | **p** |
| --- | --- | --- | --- | --- | --- |
| Movement | Intercept | 2.497 | .544 | 4.585 | **<.001** |
|  | Sex | 1.518 | .737 | 2.059 | **.043** |
|  | Age | -.080 | .034 | -2.378 | **.020** |
|  | Group | 1.378 | .833 | 1.654 | .103 |
|  | Sex*Age | .030 | .039 | .752 | .455 |
|  | Age*Group | -.009 | .039 | -.225 | .822 |
|  | Sex*Group | -1.492 | 1.116 | -1.336 | .186 |

**Table S2**: The effects of age, sex, and their interactions on head movement (groups combined).

|  | **Effect** | **B** | **SE** | **t** | **p** |
| --- | --- | --- | --- | --- | --- |
| Movement | Intercept | 3.084 | .409 | 4.542 | **<.001** |
|  | Age*Sex | .022 | .039 | .572 | .569 |
|  | Sex | .878 | .550 | 1.598 | .114 |
|  | Age | -.077 | .028 | -2.784 | **.007** |

**Table S3**: Regression results examining effects of Surgency, Age, and Sex and interactions between Sex*Surgency and Age*Surgency.

|  | **Effect** | **B** | **SE** | **t** | **p** |
| --- | --- | --- | --- | --- | --- |
| Movement | Intercept | 5.323 | 2.357 | 2.259 | **.027** |
|  | Surgency | -.286 | .477 | -.600 | .550 |
|  | Age | -.011 | .125 | -.088 | .930 |
|  | Sex | -3.653 | 3.858 | -.947 | .347 |
|  | Sex*Surgency | .594 | .808 | .734 | .465 |
|  | Age*Surgency | -.012 | .026 | -.466 | .642 |

**Table S4**: Regression results examining effects of Effortful Control, Age, Sex, and interactions.

|  | **Effect** | **B** | **SE** | **t** | **p** |
| --- | --- | --- | --- | --- | --- |
| Movement | Intercept | 10.221 | 2.695 | 3.792 | **<.001** |
|  | Effortful Control | -1.252 | .533 | -2.350 | **.022** |
|  | Age | -.010 | .155 | -.065 | .948 |
|  | Sex | -8.302 | 5.256 | -1.580 | .119 |
|  | Sex*Effortful Control | 1.469 | .997 | 1.474 | .145 |
|  | Age*Effortful Control | -.011 | .030 | -.381 | .704 |

**Table S5**: Regression results examining effects of Negative Affectivity, Age, Sex, and interactions.

|  | **Effect** | **B** | **SE** | **t** | **p** |
| --- | --- | --- | --- | --- | --- |
| Movement | Intercept | .655 | 1.994 | .328 | .744 |
|  | Negative Affectivity | .849 | .501 | 1.693 | .095 |
|  | Age | -.076 | .120 | -.629 | .532 |
|  | Sex | 4.865 | 3.036 | 1.602 | .114 |
|  | Sex*Negative Affectivity | -1.489 | .772 | -1.928 | .058 |
|  | Age*Negative Affectivity | .001 | .031 | .027 | .979 |

**.7mm results**

**Table S6**: The effects of sex, age, clinical status (stuttering, control) and their interactions on head movement.

|  | **Effect** | **B** | **SE** | **t** | **p** |
| --- | --- | --- | --- | --- | --- |
| Movement | Intercept | 3.122 | .548 | 5.694 | **<.001** |
|  | Sex | 1.973 | .742 | 2.659 | **.010** |
|  | Age | -.081 | .034 | -2.400 | **.019** |
|  | Group | .892 | .839 | 1.064 | .291 |
|  | Sex*Age | .005 | .040 | .132 | .895 |
|  | Age*Group | .015 | .040 | .381 | .705 |
|  | Sex*Group | -1.553 | 1.124 | -1.381 | .172 |

**Table S7**: The effects of age, sex, and their interactions on head movement (groups combined).

|  | **Effect** | **B** | **SE** | **t** | **p** |
| --- | --- | --- | --- | --- | --- |
| Movement | Intercept | 3.524 | .410 | 8.591 | **<.001** |
|  | Age*Sex | .001 | .039 | .024 | .981 |
|  | Sex | 1.274 | .551 | 2.310 | **.024** |
|  | Age | -.070 | .028 | -2.498 | **.015** |

**Table S8**: Regression results examining effects of Surgency, Age, and Sex and interactions between Sex*Surgency and Age*Surgency.

|  | **Effect** | **B** | **SE** | **t** | **p** |
| --- | --- | --- | --- | --- | --- |
| Movement | Intercept | 5.205 | 2.351 | 2.214 | **.030** |
|  | Surgency | -.091 | .476 | -.192 | .848 |
|  | Age | .063 | .125 | .506 | .614 |
|  | Sex | -2.214 | 3.849 | -.575 | .567 |
|  | Sex*Surgency | .221 | .807 | .274 | .785 |
|  | Age*Surgency | -.208 | .026 | -1.082 | .283 |

**Table S9**: Regression results examining effects of Effortful Control, Age, Sex, and interactions.

|  | **Effect** | **B** | **SE** | **t** | **p** |
| --- | --- | --- | --- | --- | --- |
| Movement | Intercept | 12.381 | 2.633 | 4.701 | **<.001** |
|  | Effortful Control | -1.512 | .521 | -2.904 | **.005** |
|  | Age | -.098 | .152 | -.643 | .522 |
|  | Sex | -12.235 | 5.135 | -2.383 | **.020** |
|  | Sex*Effortful Control | 2.14 | .974 | 2.199 | **.031** |
|  | Age*Effortful Control | .005 | .029 | .157 | .875 |

**Table S10**: Regression results examining effects of Negative Affectivity, Age, Sex, and interactions.

|  | **Effect** | **B** | **SE** | **t** | **P** |
| --- | --- | --- | --- | --- | --- |
| Movement | Intercept | 2.217 | 2.009 | 1.103 | .274 |
|  | Negative Affectivity | .670 | .505 | 1.327 | .189 |
|  | Age | -.047 | .121 | -.390 | .698 |
|  | Sex | 3.704 | 3.059 | 1.211 | .230 |
|  | Sex*Negative Affectivity | -1.297 | .778 | -1.666 | .100 |
|  | Age*Negative Affectivity | -.007 | .031 | -.227 | .821 |

**.3mm results**

**Table S11**: The effects of sex, age, clinical status (stuttering, control) and their interactions on head movement.

|  | **Effect** | **B** | **SE** | **t** | **p** |
| --- | --- | --- | --- | --- | --- |
| Movement | Intercept | 5.155 | .482 | 10.694 | **<.001** |
|  | Sex | 1.543 | .653 | 2.365 | **.021** |
|  | Age | -.069 | .030 | -2.317 | **.024** |
|  | Group | .761 | .738 | 1.032 | .306 |
|  | Sex*Age | -.001 | .035 | -.024 | .981 |
|  | Age*Group | .009 | .035 | .262 | .794 |
|  | Sex*Group | -1.542 | .988 | -1.560 | .123 |

**Table S12**: The effects of age, sex, and their interactions on head movement (groups combined).

|  | **Effect** | **B** | **SE** | **t** | **p** |
| --- | --- | --- | --- | --- | --- |
| Movement | Intercept | 5.494 | .362 | 15.185 | **<.001** |
|  | Age*Sex | -.005 | .035 | -.134 | .894 |
|  | Sex | .852 | .486 | 1.751 | .084 |
|  | Age | -.061 | .025 | -2.474 | **.016** |

**Table S13**: Regression results examining effects of Surgency, Age, and Sex and interactions between Sex*Surgency and Age*Surgency.

|  | **Effect** | **B** | **SE** | **t** | **p** |
| --- | --- | --- | --- | --- | --- |
| Movement | Intercept | 6.447 | 2.067 | 3.120 | **.003** |
|  | Surgency | -.028 | .418 | -.068 | .946 |
|  | Age | .077 | .110 | .705 | .483 |
|  | Sex | -1.766 | 3.383 | -.522 | .603 |
|  | Sex*Surgency | .220 | .709 | .311 | .757 |
|  | Age*Surgency | -.030 | .023 | -1.309 | .195 |

**Table S14**: Regression results examining effects of Effortful Control, Age, Sex, and interactions.

|  | **Effect** | **B** | **SE** | **t** | **p** |
| --- | --- | --- | --- | --- | --- |
| Movement | Intercept | 13.147 | 2.314 | 5.681 | **<.001** |
|  | Effortful Control | -1.355 | .457 | -2.962 | **.004** |
|  | Age | -.107 | .133 | -.805 | .423 |
|  | Sex | -10.799 | 4.512 | -2.393 | **.019** |
|  | Sex*Effortful Control | 1.942 | .856 | 2.269 | **.026** |
|  | Age*Effortful Control | .008 | .025 | .301 | .764 |

**Table S15**: Regression results examining effects of Negative Affectivity, Age, Sex, and interactions.

|  | **Effect** | **B** | **SE** | **t** | **p** |
| --- | --- | --- | --- | --- | --- |
| Movement | Intercept | 4.432 | 1.762 | 2.515 | **.014** |
|  | Negative Affectivity | .501 | .443 | 1.131 | .262 |
|  | Age | -.030 | .106 | -.282 | .779 |
|  | Sex | 4.093 | 2.682 | 1.526 | .132 |
|  | Sex*Negative Affectivity | -1.293 | .682 | -1.895 | .062 |
|  | Age*Negative Affectivity | -.010 | .027 | -.354 | .724 |
